# Left Ventricular Assist Device Implantation in a Thrombosed Apical Aneurysm

**DOI:** 10.3390/clinpract11030057

**Published:** 2021-07-01

**Authors:** Daniele Masarone, Enrico Melillo, Rita Gravino, Rossella Vastarella, Angelo Caiazzo, Fabio Ursomando, Giuseppe Pacileo, Andrea Petraio

**Affiliations:** 1 Heart Failure Cardiology Unit, Department of Cardiology, AO dei Colli, Monaldi Hospital, 80131 Naples, Italy; Enrico.melillo@ospedalideicolli.it (E.M.); ritagravino@virgilio.it (R.G.); rossellavastarella86@gmail.com (R.V.); pacileo@gmail.com (G.P.); 2 Heart Transplant Unit, Department of Cardiovascular Surgery and Transplants, AO dei Colli, Monaldi Hospital, 80131 Naples, Italy; caiazzo@gmail.com (A.C.); Fabio.ursomando@ospedalideicolli.it (F.U.); Andrea.petraio@ospedalideicolli.it (A.P.)

**Keywords:** advanced heart failure, apical aneurysm, HeartMate III, left ventricular assist device

## Abstract

Left ventricular assist device implantation is a challenging procedure in the presence of a giant thrombosed aneurysm, and no standard surgical techniques are currently recommended in this setting. In this case, we report the successful implantation of a left ventricular assist device (HeartMate III) in a patient with a massive thrombosed apical aneurysm. The patient presented with extended antero-apical necrosis as a result of a delay in hospital admission for acute coronary syndrome due to the patient’s concerns about the COVID-19 pandemic outbreak.

## 1. Introduction

Despite the improvement in heart failure (HF) management, about 1–10% of patients with HF have an advanced form of the disease, characterized by peculiar pathophysiologic characteristics and care needs [1].

A heart transplant is considered the standard of care for carefully selected patients with advanced HF; however, the left ventricular assist device (LVAD) has rapidly emerged as a durable and safe therapy for these patients [2].

In patients with ischemic dilated cardiomyopathy and thrombosed left ventricular apical aneurysm, LVAD implantation represents a challenging issue. The main concerns are related to myocardial wall rupture (due to the thinning of the left ventricular wall), cerebrovascular events (due to potential mobilisation of thrombosed material) and the possibility of non-optimal position and alignment of the inflow cannula (due to complex surgical anatomy).

Furthermore, there are no clear international recommendations for the treatment of these patients, and only a single case report describing LVAD implantation combined with ventricular surgical reconstruction in a patient with advanced HF and left ventricular aneurysm has been previously published [3].

In the present case, we describe a successful HeartMate III (Abbott Laboratories, Abbott Park, IL, USA) LVAD implantation in a patient with ischemic dilated cardiomyopathy and an extended thrombosed antero-apical ischaemic aneurysm.

## 2. Case Report

**Figure 1:**
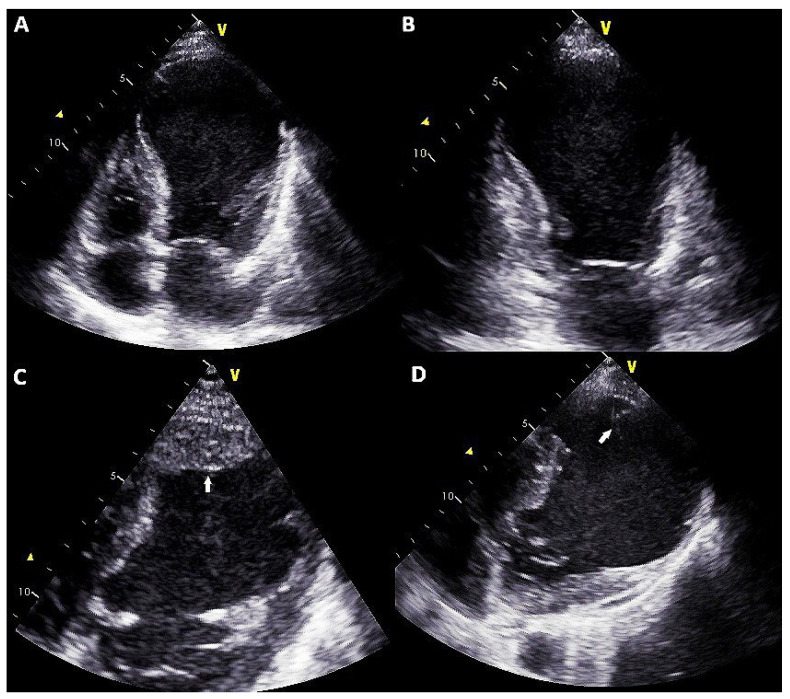
Transthoracic echocardiography four-chamber (**A**) and two-chamber (**B**) views showing apical aneurysm of the left ventricle. Focused off-axis view of the apical region showing thrombotic stratification (**C**) evolved into pedunculated and mobile thrombotic material (**D**) after anticoagulant treatment.

A 50-year-old man with a medical history of smoking, hypertension and depression was admitted to our unit for acute HF in May 2020. In March 2020, he referred several angina episodes with no hospital admission due to fear of the COVID-19 pandemic. The electrocardiogram at admission showed signs of extended anteroseptal necrosis. Echocardiography showed a massive thrombosed antero-apical left ventricular aneurysm and severely impaired left ventricular systolic function (left ventricular ejection fraction of 15%) and normal right ventricle systolic function (Figure 1A–D). Coronary angiography revealed a chronic occlusion of the left anterior descending artery, with no indication for percutaneous unclogging due to the absence of myocardial ischemia and viability. A cardiac magnetic resonance imaging showed severe eccentric hypertrophy of the left ventricle (left ventricular mass index 198 g/m^2^, relative wall thickness 0.27, left ventricular end-diastolic volume 307 mL; left ventricular end-systolic volume 265 mL), with evidence of thrombosed apical aneurysm.

A right heart catheterization revealed post-capillary pulmonary hypertension (mean pulmonary pressure 25 mmHg, wedge pressure 20 mmHg), with normal pulmonary vascular resistance (pulmonary vascular resistance 1.3 woods units) and reducing systolic function (cardiac output 3.8 L/min; cardiac index 2 L/min/m^2^).

Despite intensive diuretic and inotropic treatments, the patient’s diuresis did not improve (mean 24 h diuresis of the first three days 1200 mL); at four days post admission, acute kidney and liver injury persisted and signs and symptoms of low cardiac output syndrome appeared. (Table 1).

Moreover, despite proper anticoagulant therapy with warfarin, the patient experienced multiple cardio-embolic ischemic transient attacks.

Considered the clinic status of the patient (INTERMACS class III, left ventricular ejection fraction <25% despite the infusion of diuretics and inotropes), the shared decision of the heart team, in agreement to the European Association for Cardio-Thoracic Surgery Expert Consensus Document on long-term mechanical circulatory support [4], was to perform HeartMate III implantation.

**Figure 2:**
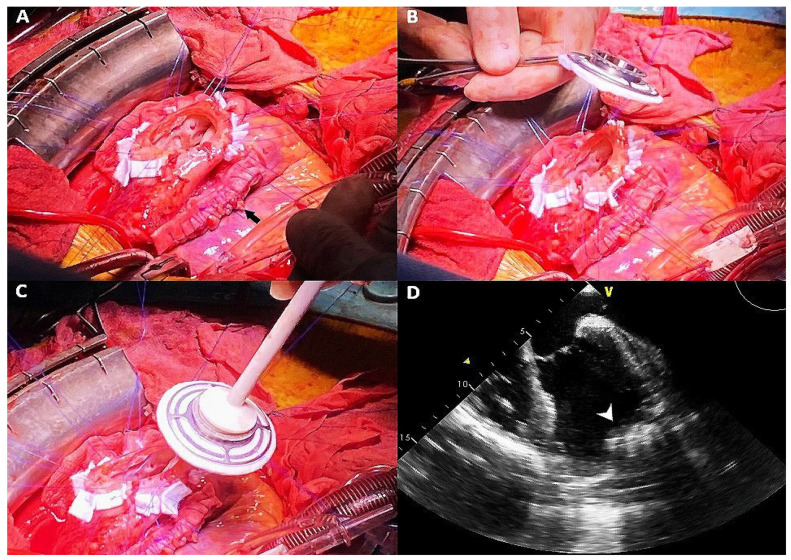
Surgical placement of left ventricular assist device: after performing left ventricle external plication (black arrow) and incision of the apical aneurysm (**A**), a sewing ring was placed in the apical region (**B**,**C**), resulting in a more lateral position of the inflow cannula ((**D**), transoesophageal echocardiography, white arrowhead).

The surgical procedure was performed with median sternotomy, a traditional cardiopulmonary bypass, clamping of the aorta and anterograde cardioplegic arrest. Surgical inspection revealed an extended pseudoaneurysm of the anterolateral left ventricular wall, which was treated with a Jatene-like external plication technique, employing a double-layer suture from one side of the pseudoaneurysm to the other and 3-0 prolene sutures with strips of Teflon to restore the left ventricular anterolateral wall shape (Figure 2A). After incision of the apical aneurysm parallel to the left descending coronary artery, the ventricular cavity was inspected, and residual thrombotic material adherent to the subendocardial wall was removed.

Considering the fibrous scar and thinning of the apex, we chose to place the inflow cannula in the anterolateral side of the left ventricular apex, maintaining an alignment parallel to the interventricular septum. The sewing ring was secured with twelve 3-0 prolene stitches and reinforced with pledgets. After placing the inflow cannula in the ventricular cavity and anastomosis of the outflow graft with the ascending aorta, the driveline was tunnelled and connected to the external unit.

The patient was successfully weaned from mechanical ventilation on the first postoperative day without inotropic support. In addition, no significant complications occurred during the postoperative course. The patient was discharged six weeks after surgery, with no resting or exertional dyspnoea (New York Heart Association class I).

At one year follow-up, the patient reported no hospitalizations for HF, stable New York Heart Association class I and few LVAD alarms with a high pulsatility index (a measurement of the flow pulse through the pump) due to low preload and elevated mean arterial blood pressure, resolved, respectively, through withdrawal of diuretic therapy and progressive up-titration of lisinopril and amlodipine.

## 3. Conclusions

Left ventricular apical aneurysm due to ischemic cardiomyopathy is a surgical challenge for HF patients, potentially requiring LVAD therapy.

In the present report, LVAD placement and concomitant surgical plication of the anterolateral wall pseudoaneurysm were successfully performed, and the patient experienced an uncomplicated postoperative course with an excellent clinical outcome. Therefore, in the presence of a left apical aneurysm, LVAD implantation and eventual concomitant LV surgical reconstruction are feasible therapeutic options and should be tailored for each patient.

## Figures and Tables

**Table 1:** Timetable of the clinical case.

March 2020	Angina Episodes
12 May 2020	Hospitalization for acute heart failure with diagnosis of ischemic dilated cardiomyopathy with severe reduction of ejection fraction and thrombosed apical aneurysm Clinical presentation was complicated by acute kidney injury (eGFR at admission 38 mL/min/m^2^ vs. 57 mL/min/m^2^ of March 2020) and acute liver injury (biluribin 3.5 mg/dL, AST 560 UI/L, ALT 654 UI/L) High dose diuretics therapy (furosemide 250 mg bid plus spironolactone 200 mg/die) and levosimendan infusion was started (0.1 mcg/kg/min)
13 May 2020	24 h diuresis 1100 mL; metolazone (5 mg bid) was added
15 May 2020	24 h diuresis 1300 mL; enoximone infusion (5 mcg/kg/min) was started. Noradrenaline infusion (0.2 mcg/kg/min) was started. Episode of cardioembolic transitory ischemic attack occurs
16 May 2020	24 h diuresis 1200 mL. Episode of cardioembolic transitory ischemic attack occurs
17 May 2020	Low cardiac output syndrome appears Heart team reunion and indication to LVAD
18 May 2020	Heartmate III implantation with and concomitant surgical plication of the anterolateral wall pseudoaneurysm
28 June 2020	Patient discharged
02 May 2021	At follow-up non hospitalizations for heart failure and stable NYHA class

## Data Availability

Not applicable.
